# Novel risk loci for COVID-19 hospitalization among admixed American populations

**DOI:** 10.7554/eLife.93666

**Published:** 2024-10-03

**Authors:** Silvia Diz-de Almeida, Raquel Cruz, Andre D Luchessi, José M Lorenzo-Salazar, Miguel López de Heredia, Inés Quintela, Rafaela González-Montelongo, Vivian Nogueira Silbiger, Marta Sevilla Porras, Jair Antonio Tenorio Castaño, Julian Nevado, Jose María Aguado, Carlos Aguilar, Sergio Aguilera-Albesa, Virginia Almadana, Berta Almoguera, Nuria Alvarez, Álvaro Andreu-Bernabeu, Eunate Arana-Arri, Celso Arango, María J Arranz, Maria-Jesus Artiga, Raúl C Baptista-Rosas, María Barreda- Sánchez, Moncef Belhassen-Garcia, Joao F Bezerra, Marcos AC Bezerra, Lucía Boix-Palop, María Brion, Ramón Brugada, Matilde Bustos, Enrique J Calderón, Cristina Carbonell, Luis Castano, Jose E Castelao, Rosa Conde-Vicente, M Lourdes Cordero-Lorenzana, Jose L Cortes-Sanchez, Marta Corton, M Teresa Darnaude, Alba De Martino-Rodríguez, Victor del Campo-Pérez, Aranzazu Diaz de Bustamante, Elena Domínguez-Garrido, Rocío Eirós, María Carmen Fariñas, María J Fernandez-Nestosa, Uxía Fernández-Robelo, Amanda Fernández-Rodríguez, Tania Fernández-Villa, Manuela Gago-Dominguez, Belén Gil-Fournier, Javier Gómez-Arrue, Beatriz González Álvarez, Fernan Gonzalez Bernaldo de Quirós, Anna González-Neira, Javier González-Peñas, Juan F Gutiérrez-Bautista, María José Herrero, Antonio Herrero-Gonzalez, María A Jimenez-Sousa, María Claudia Lattig, Anabel Liger Borja, Rosario Lopez-Rodriguez, Esther Mancebo, Caridad Martín-López, Vicente Martín, Oscar Martinez-Nieto, Iciar Martinez-Lopez, Michel F Martinez-Resendez, Angel Martinez-Perez, Juliana F Mazzeu, Eleuterio Merayo Macías, Pablo Minguez, Victor Moreno Cuerda, Silviene F Oliveira, Eva Ortega-Paino, Mara Parellada, Estela Paz-Artal, Ney PC Santos, Patricia Pérez-Matute, Patricia Perez, M Elena Pérez-Tomás, Teresa Perucho, Mellina Pinsach-Abuin, Guillermo Pita, Ericka N Pompa-Mera, Gloria L Porras-Hurtado, Aurora Pujol, Soraya Ramiro León, Salvador Resino, Marianne R Fernandes, Emilio Rodríguez-Ruiz, Fernando Rodriguez-Artalejo, José A Rodriguez-Garcia, Francisco Ruiz-Cabello, Javier Ruiz-Hornillos, Pablo Ryan, José Manuel Soria, Juan Carlos Souto, Eduardo Tamayo, Alvaro Tamayo-Velasco, Juan Carlos Taracido-Fernandez, Alejandro Teper, Lilian Torres-Tobar, Miguel Urioste, Juan Valencia-Ramos, Zuleima Yáñez, Ruth Zarate, Itziar de Rojas, Agustín Ruiz, Pascual Sánchez, Luis Miguel Real, SCOURGE Cohort Group, Encarna Guillen-Navarro, Carmen Ayuso, Esteban Parra, José A Riancho, Augusto Rojas-Martinez, Carlos Flores, Pablo Lapunzina, Ángel Carracedo

**Affiliations:** 1 ERN-ITHACA-European Reference Network Soria Spain; 2 Pediatric Neurology Unit, Department of Pediatrics, Navarra Health Service Hospital Pamplona Spain; 3 CIBERER, ISCIII Madrid Spain; 4 Centro Singular de Investigación en Medicina Molecular y Enfermedades Crónicas (CIMUS), Universidade de Santiago de Compostela Santiago de Compostela Spain; 5 Universidade Federal do Rio Grande do Norte, Departamento de Analises Clinicas e Toxicologicas Natal Brazil; 6 Genomics Division, Instituto Tecnológico y de Energías Renovables Santa Cruz de Tenerife Spain; 7 Fundación Pública Galega de Medicina Xenómica, Sistema Galego de Saúde (SERGAS) Santiago de Compostela Spain; 8 Instituto de Genética Médica y Molecular (INGEMM), Hospital Universitario La Paz IDIPAZ Madrid Spain; 9 Unit of Infectious Diseases, Hospital Universitario 12 de Octubre, Instituto de Investigación Sanitaria Hospital 12 de Octubre (imas12) Madrid Spain; 10 Spanish Network for Research in Infectious Diseases (REIPI RD16/0016/0002), Instituto de Salud Carlos III Madrid Spain; 11 CIBERINFEC, ISCIII Madrid Spain; 12 Hospital General Santa Bárbara de Soria Soria Spain; 13 Navarra Health Service, NavarraBioMed Research Group Pamplona Spain; 14 Hospital Universitario Virgen Macarena, Neumología Sevilla Spain; 15 Department of Genetics & Genomics, Instituto de Investigación Sanitaria-Fundación Jiménez Díaz University Hospital - Universidad Autónoma de Madrid (IIS-FJD, UAM) Madrid Spain; 16 Spanish National Cancer Research Centre, Human Genotyping-CEGEN Unit Madrid Spain; 17 Department of Child and Adolescent Psychiatry, Institute of Psychiatry and Mental Health, Hospital General Universitario Gregorio Marañón (IiSGM) Madrid Spain; 18 School of Medicine, Universidad Complutense Madrid Spain; 19 Biocruces Bizkai HRI Bizkaia Spain; 20 Cruces University Hospital, Osakidetza Bizkaia Spain; 21 Centre for Biomedical Network Research on Mental Health (CIBERSAM), Instituto de Salud Carlos III Madrid Spain; 22 Fundació Docència I Recerca Mutua Terrassa Barcelona Spain; 23 Spanish National Cancer Research Centre, CNIO Biobank Madrid Spain; 24 Hospital General de Occidente Zapopan Jalisco Mexico; 25 Centro Universitario de Tonalá, Universidad de Guadalajara Tonalá Jalisco Mexico; 26 Centro de Investigación Multidisciplinario en Salud, Universidad de Guadalajara Tonalá Jalisco Mexico; 27 Universidad Católica San Antonio de Murcia (UCAM) Murcia Spain; 28 Instituto Murciano de Investigación Biosanitaria (IMIB-Arrixaca) Murcia Spain; 29 Hospital Universitario de Salamanca-IBSAL, Servicio de Medicina Interna-Unidad de Enfermedades Infecciosas Salamanca Spain; 30 Escola Tecnica de Saúde, Laboratorio de Vigilancia Molecular Aplicada Brasilia Brazil; 31 Federal University of Pernambuco, Genetics Postgraduate Program Recife Brazil; 32 Hospital Universitario Mutua Terrassa Barcelona Spain; 33 Instituto de Investigación Sanitaria de Santiago (IDIS), Xenética Cardiovascular Santiago de Compostela Spain; 34 CIBERCV, ISCIII Madrid Spain; 35 Cardiovascular Genetics Center, Institut d’Investigació Biomèdica Girona (IDIBGI) Girona Spain; 36 Medical Science Department, School of Medicine, University of Girona Girona Spain; 37 Hospital Josep Trueta, Cardiology Service Girona Spain; 38 Institute of Biomedicine of Seville (IBiS), Consejo Superior de Investigaciones Científicas (CSIC)- University of Seville- Virgen del Rocio University Hospital Seville Spain; 39 Departamento de Medicina, Hospital Universitario Virgen del Rocío, Universidad de Sevilla Seville Spain; 40 CIBERESP, ISCIII Madrid Spain; 41 Hospital Universitario de Salamanca-IBSAL, Servicio de Medicina Interna Salamanca Spain; 42 Universidad de Salamanca Salamanca Spain; 43 Osakidetza, Cruces University Hospital Bizkaia Spain; 44 Centre for Biomedical Network Research on Diabetes and Metabolic Associated Diseases (CIBERDEM), Instituto de Salud Carlos III Madrid Spain; 45 University of Pais Vasco, UPV/EHU Bizkaia Spain; 46 Oncology and Genetics Unit, Instituto de Investigacion Sanitaria Galicia Sur, Xerencia de Xestion Integrada de Vigo-Servizo Galego de Saúde Vigo Spain; 47 Hospital Universitario Río Hortega Valladolid Spain; 48 Servicio de Medicina intensiva, Complejo Hospitalario Universitario de A Coruña (CHUAC), Sistema Galego de Saúde (SERGAS) A Coruña Spain; 49 Tecnológico de Monterrey Monterrey Mexico; 50 Department of Microgravity and Translational Regenerative Medicine, Otto von Guericke University Magdeburg Germany; 51 Hospital Universitario Mostoles, Unidad de Genética Madrid Spain; 52 Instituto Aragonés de Ciencias de la Salud (IACS) Zaragoza Spain; 53 Instituto Investigación Sanitaria Aragón (IIS-Aragon) Zaragoza Spain; 54 Preventive Medicine Department, Instituto de Investigacion Sanitaria Galicia Sur, Xerencia de Xestion Integrada de Vigo-Servizo Galego de Saúde Vigo Spain; 55 Unidad Diagnóstico Molecular, Fundación Rioja Salud La Rioja Spain; 56 Hospital Universitario de Salamanca-IBSAL, Servicio de Cardiología Salamanca Spain; 57 IDIVAL Cantabria Spain; 58 Hospital U M Valdecilla Cantabria Spain; 59 Universidad de Cantabria Cantabria Spain; 60 Universidad Nacional de Asunción, Facultad de Politécnica Paraguay United States; 61 Urgencias Hospitalarias, Complejo Hospitalario Universitario de A Coruña (CHUAC), Sistema Galego de Saúde (SERGAS) A Coruña Spain; 62 Unidad de Infección Viral e Inmunidad, Centro Nacional de Microbiología (CNM), Instituto de Salud Carlos III (ISCIII) Madrid Spain; 63 Grupo de Investigación en Interacciones Gen-Ambiente y Salud (GIIGAS) - Instituto de Biomedicina (IBIOMED), Universidad de León León Spain; 64 IDIS Seongnam Republic of Korea; 65 Hospital Universitario de Getafe, Servicio de Genética Madrid Spain; 66 Ministerio de Salud Ciudad de Buenos Aires Buenos Aires Argentina; 67 Hospital Universitario Virgen de las Nieves, Servicio de Análisis Clínicos e Inmunología Granada Spain; 68 IIS La Fe, Plataforma de Farmacogenética Valencia Spain; 69 Universidad de Valencia, Departamento de Farmacología Valencia Spain; 70 Data Analysis Department, Instituto de Investigación Sanitaria-Fundación Jiménez Díaz University Hospital - Universidad Autónoma de Madrid (IIS-FJD, UAM) Madrid Spain; 71 Universidad de los Andes, Facultad de Ciencias Bogotá Colombia; 72 SIGEN Alianza Universidad de los Andes - Fundación Santa Fe de Bogotá Bogotá Colombia; 73 Hospital General de Segovia, Medicina Intensiva Segovia Spain; 74 Facultad de Farmacia, Universidad San Pablo-CEU, CEU Universities, Urbanización Montepríncipe Boadilla del Monte Spain; 75 Hospital Universitario 12 de Octubre, Department of Immunology Madrid Spain; 76 Instituto de Investigación Sanitaria Hospital 12 de Octubre (imas12), Transplant Immunology and Immunodeficiencies Group Madrid Spain; 77 Fundación Santa Fe de Bogota, Departamento Patologia y Laboratorios Bogotá Colombia; 78 Unidad de Genética y Genómica Islas Baleares Islas Baleares Spain; 79 Hospital Universitario Son Espases, Unidad de Diagnóstico Molecular y Genética Clínica Islas Baleares Spain; 80 Genomics of Complex Diseases Unit, Research Institute of Hospital de la Santa Creu i Sant Pau, IIB Sant Pau Barcelona Spain; 81 Universidade de Brasília, Faculdade de Medicina Brasília Brazil; 82 Programa de Pós-Graduação em Ciências Médicas (UnB) Brasília Brazil; 83 Programa de Pós-Graduação em Ciencias da Saude (UnB) Brazila Brazil; 84 Hospital El Bierzo, Unidad Cuidados Intensivos León Spain; 85 Hospital Universitario Mostoles, Medicina Interna Madrid, Spai Spain; 86 Universidad Francisco de Vitoria Madrid Spain; 87 Departamento de Genética e Morfologia, Instituto de Ciências Biológicas, Universidade de Brasília Brasília Brazil; 88 Programa de Pós-Graduação em Biologia Animal (UnB) Brasília Brazil; 89 Programa de Pós-Graduação Profissional em Ensino de Biologia (UnB) Brasília Brazil; 90 Universidad Complutense de Madrid, Department of Immunology, Ophthalmology and ENT Madrid Spain; 91 Universidade Federal do Pará, Núcleo de Pesquisas em Oncologia Belém Brazil; 92 Infectious Diseases, Microbiota and Metabolism Unit, CSIC Associated Unit, Center for Biomedical Research of La Rioja (CIBIR) Logroño Spain; 93 Inditex, A Coruña Corunna Spain; 94 GENYCA Madrid Spain; 95 Instituto Mexicano del Seguro Social (IMSS), Centro Médico Nacional Siglo XXI, Unidad de Investigación Médica en Enfermedades Infecciosas y Parasitarias Mexico City Mexico; 96 Instituto Mexicano del Seguro Social (IMSS), Centro Médico Nacional La Raza, Hospital de Infectología Mexico City Mexico; 97 Clinica Comfamiliar Risaralda Pereira Colombia; 98 Bellvitge Biomedical Research Institute (IDIBELL), Neurometabolic Diseases Laboratory, L’Hospitalet de Llobregat Barcelona Spain; 99 Catalan Institution of Research and Advanced Studies (ICREA) Barcelona Spain; 100 Hospital Ophir Loyola, Departamento de Ensino e Pesquisa Belém Brazil; 101 Unidad de Cuidados Intensivos, Hospital Clínico Universitario de Santiago (CHUS), Sistema Galego de Saúde (SERGAS) Santiago de Compostela Spain; 102 Department of Preventive Medicine and Public Health, School of Medicine, Universidad Autónoma de Madrid Madrid Spain; 103 IdiPaz (Instituto de Investigación Sanitaria Hospital Universitario La Paz) Madrid Spain; 104 IMDEA-Food Institute, CEI UAM+CSIC Madrid Spain; 105 Complejo Asistencial Universitario de León León Spain; 106 Instituto de Investigación Biosanitaria de Granada (ibs GRANADA) Granada Spain; 107 Universidad de Granada, Departamento Bioquímica, Biología Molecular e Inmunología III Granada Spain; 108 Hospital Infanta Elena, Allergy Unit, Valdemoro Madrid Spain; 109 Instituto de Investigación Sanitaria-Fundación Jiménez Díaz University Hospital - Universidad Autónoma de Madrid (IIS-FJD, UAM) Madrid Spain; 110 Faculty of Medicine, Universidad Francisco de Vitoria Madrid Spain; 111 Hospital Universitario Infanta Leonor Madrid Spain; 112 Complutense University of Madrid Madrid Spain; 113 Gregorio Marañón Health Research Institute (IiSGM) Madrid Spain; 114 Haemostasis and Thrombosis Unit, Hospital de la Santa Creu i Sant Pau, IIB Sant Pau Barcelona Spain; 115 Hospital Clinico Universitario de Valladolid, Servicio de Anestesiologia y Reanimación Valladolid Spain; 116 Universidad de Valladolid, Departamento de Cirugía Valladolid Spain; 117 Hospital Clinico Universitario de Valladolid, Servicio de Hematologia y Hemoterapia Valladolid Spain; 118 Hospital de Niños Ricardo Gutierrez Buenos Aires Argentina; 119 Fundación Universitaria de Ciencias de la Salud Bogotá Colombia; 120 Spanish National Cancer Research Centre, Familial Cancer Clinical Unit Madrid Spain; 121 University Hospital of Burgos Burgos Spain; 122 Universidad Simón Bolívar, Facultad de Ciencias de la Salud Barranquilla Colombia; 123 Centro para el Desarrollo de la Investigación Científica Asunción Paraguay; 124 Centre for Biomedical Network Research on Neurodegenerative Diseases (CIBERNED), Instituto de Salud Carlos III Madrid Spain; 125 Research Center and Memory clinic, ACE Alzheimer Center Barcelona, Universitat Internacional de Catalunya Barcelona Spain; 126 CIEN Foundation/Queen Sofia Foundation Alzheimer Center Madrid Spain; 127 Hospital Universitario de Valme, Unidad Clínica de Enfermedades Infecciosas y Microbiología Sevilla Spain; 128 Sección Genética Médica - Servicio de Pediatría, Hospital Clínico Universitario Virgen de la Arrixaca, Servicio Murciano de Salud Murcia Spain; 129 Departamento Cirugía, Pediatría, Obstetricia y Ginecología, Facultad de Medicina, Universidad de Murcia (UMU) Murcia Spain; 130 Grupo Clínico Vinculado, Centre for Biomedical Network Research on Rare Diseases (CIBERER), Instituto de Salud Carlos III Madrid Spain; 131 Department of Anthropology, University of Toronto at Mississauga Mississauga Canada; 132 Tecnologico de Monterrey, Escuela de Medicina y Ciencias de la Salud Monterrey Mexico; 133 Research Unit, Hospital Universitario Nuestra Señora de Candelaria, Instituto de Investigación Sanitaria de Canarias Santa Cruz de Tenerife Spain; 134 Department of Clinical Sciences, University Fernando Pessoa Canarias Las Palmas de Gran Canaria Spain; 135 Centre for Biomedical Network Research on Respiratory Diseases (CIBERES), Instituto de Salud Carlos III Madrid Spain; Dartmouth College United States; Seoul National University Republic of Korea

**Keywords:** GWAS, COVID-19, SNP, None

## Abstract

The genetic basis of severe COVID-19 has been thoroughly studied, and many genetic risk factors shared between populations have been identified. However, reduced sample sizes from non-European groups have limited the discovery of population-specific common risk loci. In this second study nested in the SCOURGE consortium, we conducted a genome-wide association study (GWAS) for COVID-19 hospitalization in admixed Americans, comprising a total of 4702 hospitalized cases recruited by SCOURGE and seven other participating studies in the COVID-19 Host Genetic Initiative. We identified four genome-wide significant associations, two of which constitute novel loci and were first discovered in Latin American populations (*BAZ2B* and *DDIAS*). A trans-ethnic meta-analysis revealed another novel cross-population risk locus in *CREBBP*. Finally, we assessed the performance of a cross-ancestry polygenic risk score in the SCOURGE admixed American cohort. This study constitutes the largest GWAS for COVID-19 hospitalization in admixed Latin Americans conducted to date. This allowed to reveal novel risk loci and emphasize the need of considering the diversity of populations in genomic research.

## Introduction

To date, more than 50 loci associated with COVID-19 susceptibility, hospitalization, and severity have been identified using genome-wide association studies (GWAS) (Kanai et al., 2023; Pairo-Castineira et al., 2023). The COVID-19 Host Genetics Initiative (HGI) has made significant efforts (Niemi et al., 2021) to augment the power to identify disease loci by recruiting individuals from diverse populations and conducting a trans-ancestry meta-analysis. Despite this, the lack of genetic diversity and a focus on cases of European ancestries still predominate in the studies (Popejoy and Fullerton, 2016; Sirugo et al., 2019). In addition, while trans-ancestry meta-analyses are a powerful approach for discovering shared genetic risk variants with similar effects across populations (Li and Keating, 2014), they may fail to identify risk variants that have larger effects on particular underrepresented populations. Genetic disease risk has been shaped by the particular evolutionary history of populations and environmental exposures (Rosenberg et al., 2010). Their action is particularly important for infectious diseases due to the selective constraints that are imposed by host‒pathogen interactions (Karlsson et al., 2014; Kwok et al., 2021). Literature examples of this in COVID-19 severity include a *DOCK2* gene variant in East Asians (Namkoong et al., 2022) and frequent loss-of-function variants in *IFNAR1* and *IFNAR2* genes in Polynesian and Inuit populations, respectively (Bastard et al., 2022; Duncan et al., 2022).

Including diverse populations in case‒control GWAS with unrelated participants usually requires a prior classification of individuals in genetically homogeneous groups, which are typically analyzed separately to control the population stratification effects (Peterson et al., 2019). Populations with recent admixture impose an additional challenge to GWASs due to their complex genetic diversity and linkage disequilibrium (LD) patterns, requiring the development of alternative approaches and a careful inspection of results to reduce false positives due to population structure (Rosenberg et al., 2010). In fact, there are benefits in study power from modeling the admixed ancestries either locally, at the regional scale in the chromosomes, or globally, across the genome, depending on factors such as the heterogeneity of the risk variant in frequencies or the effects among the ancestry strata (Mester et al., 2023). Despite the development of novel methods specifically tailored for the analysis of admixed populations (Atkinson et al., 2021), the lack of a standardized analysis framework and the difficulties in confidently clustering admixed individuals into particular genetic groups often lead to their exclusion from GWAS.

The Spanish Coalition to Unlock Research on Host Genetics on COVID-19 (SCOURGE) recruited COVID-19 patients between March and December 2020 from hospitals across Spain and from March 2020 to July 2021 in Latin America (https://www.scourge-covid.org). A first GWAS of COVID-19 severity among Spanish patients of European descent revealed novel disease loci and explored age- and sex-varying effects of genetic factors (Cruz et al., 2022). Here, we present the findings of a GWAS meta-analysis in admixed Latin American (AMR) populations, comprising individuals from the SCOURGE Latin American cohort and the HGI studies, which allowed us to identify two novel severe COVID-19 loci, *BAZ2B* and *DDIAS*. Further analyses modeling the admixture from three genetic ancestral components and performing a trans-ethnic meta-analysis led to the identification of an additional risk locus near *CREBBP*. We finally assessed a cross-ancestry polygenic risk score (PGS) model with variants associated with critical COVID-19.

## Results

### Meta-analysis of COVID-19 hospitalization in admixed Americans

#### Study cohorts

**Figure 1.**
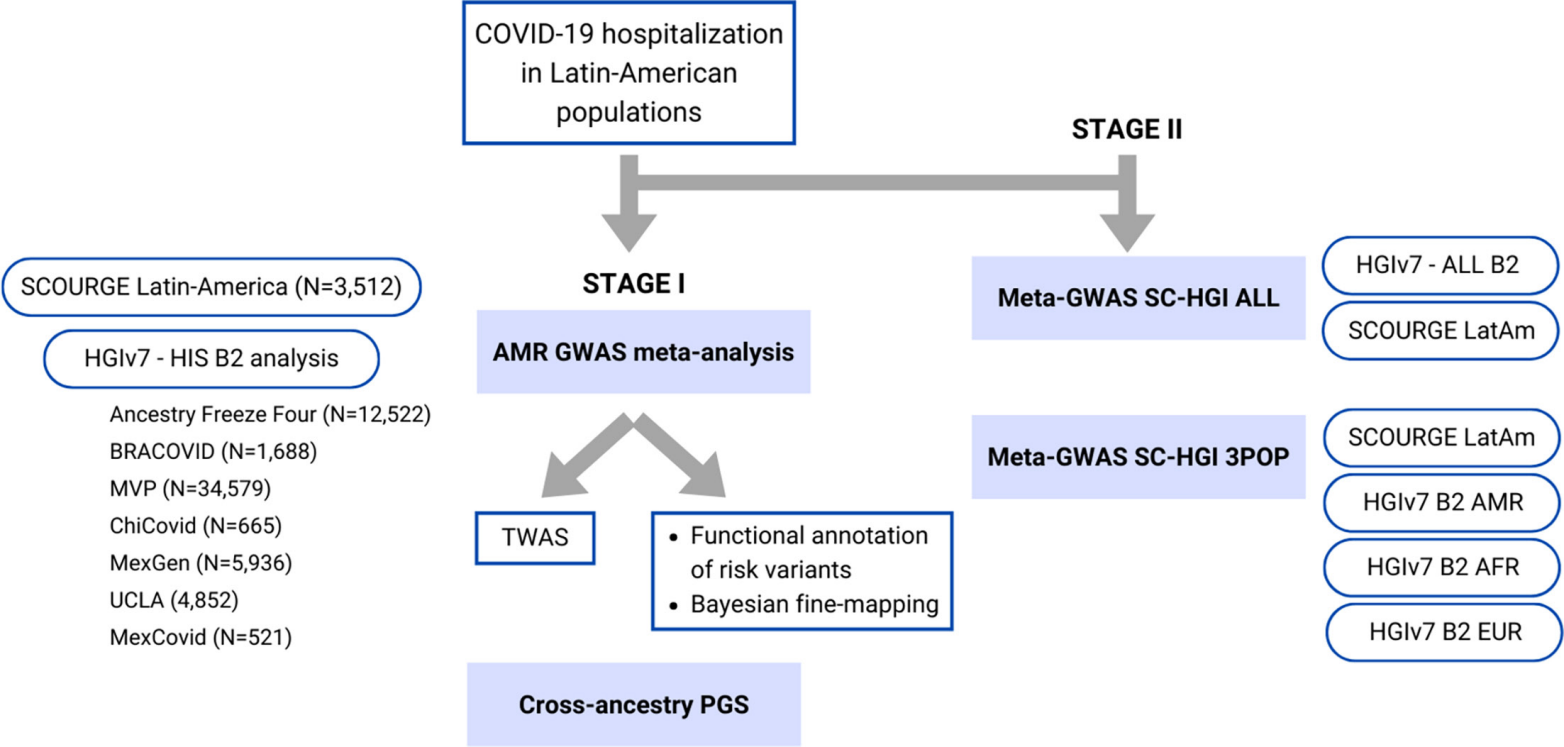
Flow chart of this study. Stage I of the study involved a meta-analysis of the Latin American genome-wide association studies (GWAS) from SCOURGE and the COVID-19 Host Genetics Initiative. The resulting meta-analysis was leveraged to prioritize genes by using a transcriptome-wide association study (TWAS), Bayesian fine-mapping and functional annotations, and to assess the generalizability of polygenic risk score (PGS) cross-population models in Latin Americans. Stage II involved two additional cross-population GWAS meta-analyses to further investigate the replicability of findings.

Within the SCOURGE consortium, we included 1608 hospitalized cases and 1887 controls (not hospitalized COVID-19 patients) from Latin American countries and from recruitments of individuals of Latin American descent conducted in Spain (Supplementary file 1). Quality control details and estimation of global genetic inferred ancestry (GIA) (Figure 1—figure supplement 1) are described in ‘Materials and methods’, whereas clinical and demographic characteristics of patients included in the analysis are shown in Table 1. Summary statistics from the SCOURGE cohort were obtained under a logistic mixed model with the SAIGE model (‘Materials and methods’). Another seven studies participating in the COVID-19 HGI consortium were included in the meta-analysis of COVID-19 hospitalization in admixed Americans (Figure 1).

**Figure 1—figure supplement 1.**
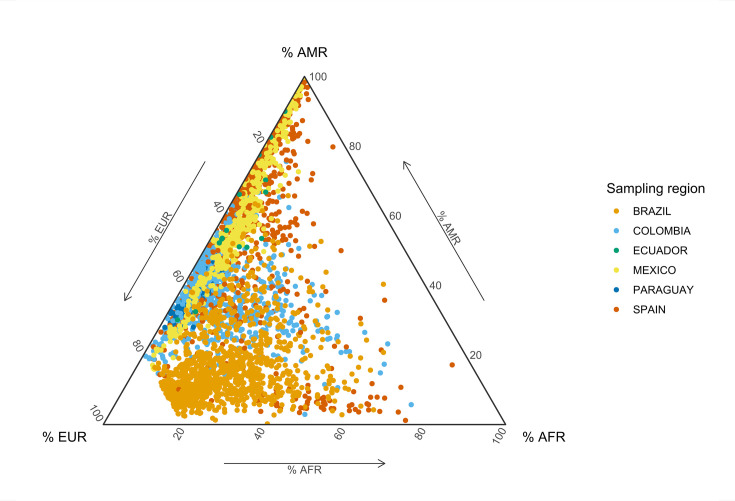
Global genetic inferred ancestry (GIA) composition in the SCOURGE Latin American cohort. European (EUR), African (AFR), and Native American (AMR) GIA was derived with ADMIXTURE from a reference panel composed of Aymaran, Mayan, Nahuan, and Quechuan individuals of Native American genetic ancestry and randomly selected samples from the EUR and AFR 1KGP populations. The colors represent the different geographical sampling regions from which the admixed American individuals from SCOURGE were recruited.

**Table 1.** Demographic characteristics of the SCOURGE Latin American cohort.

Variable		Non-hospitalized (N = 1887)	Hospitalized(N = 1625)
Age, mean years ±SD		39.1 ± 11.9	54.1 ± 14.5
Sex, N (%)			
	Female (%)	1253 (66.4)	668 (41.1)
Global genetic inferred ancestry, % mean ± SD			
	European	54.4 ± 16.2	39.4 ± 20.7
	African	15.3 ± 12.7	9.1 ± 11.6
	Native American	30.3 ± 19.8	51.3 ± 26.5
Comorbidities, N (%)			
	Vascular/endocrinological	488 (25.9)	888 (64.5)
	Cardiac	60 (3.2)	151 (9.3)
	Nervous	15 (0.8)	61 (3.8)
	Digestive	14 (0.7)	33 (2.0)
	Onco-hematological	21 (1.1)	48 (3.00)
	Respiratory	76 (4.0)	118 (7.3)

#### GWAS meta-analysis

We performed a fixed-effects GWAS meta-analysis using the inverse of the variance as weights for the overlapping markers. The combined GWAS sample size consisted of 4702 admixed AMR hospitalized cases and 68,573 controls.

**Figure 2.**
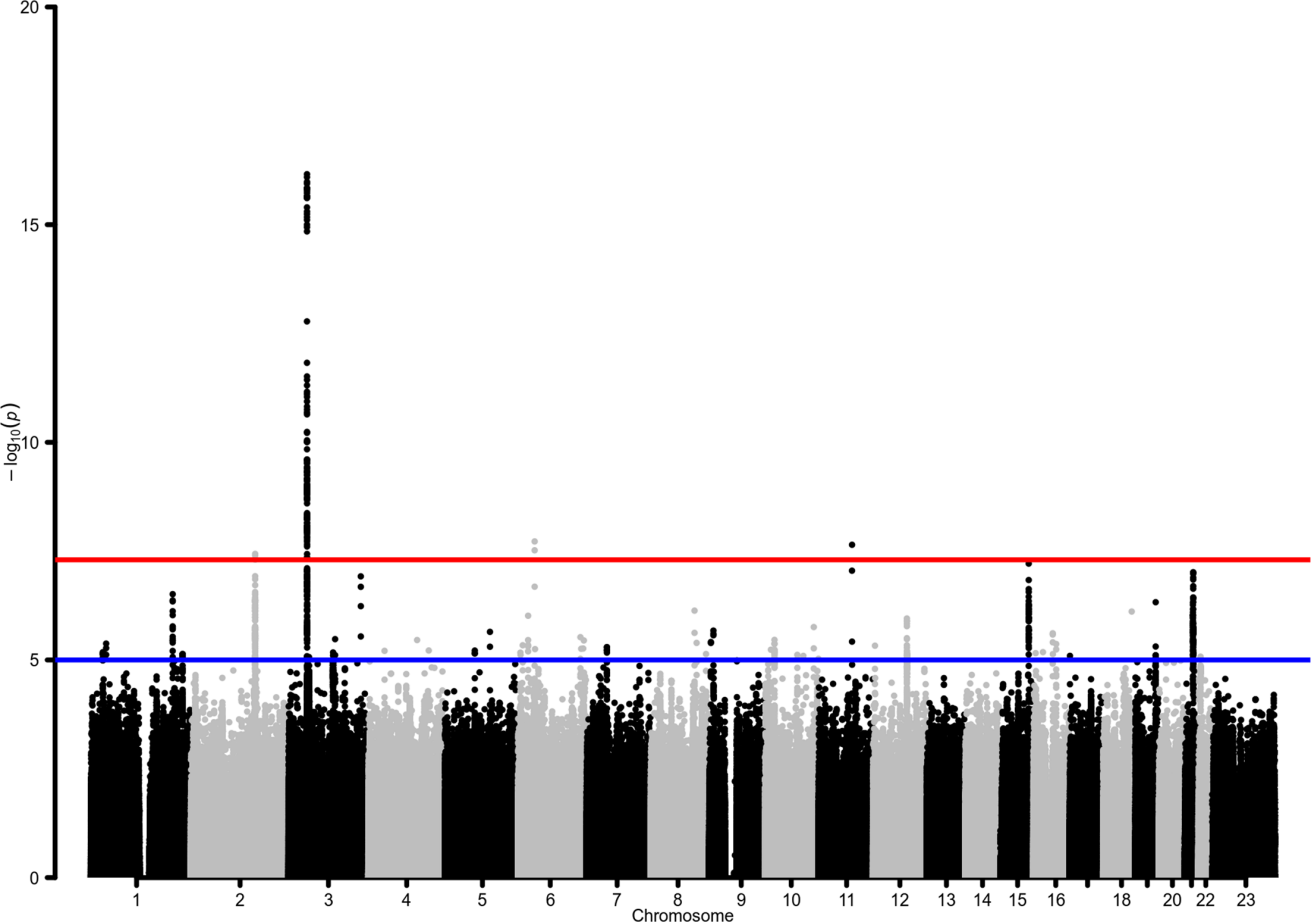
Manhattan plot for the admixed AMR genome-wide association studies (GWAS) meta-analysis. Probability thresholds at p=5 × 10^–8^ and p=5 × 10^–5^ are indicated by the horizontal lines. Genome-wide significant associations with COVID-19 hospitalizations were found on chromosome 2 (within *BAZ2B*), chromosome 3 (within *LZTFL1*), chromosome 6 (within *FOXP4*), and chromosome 11 (within *DDIAS*).

This GWAS meta-analysis revealed genome-wide significant associations at four risk loci (Table 2, Figure 2; a quantile‒quantile plot is shown in Figure 2—figure supplement 1), two of which (*BAZ2B* and *DDIAS*) were novel discoveries. A Meta-Analysis Model-based Assessment of replicability (MAMBA) approach to leverage the strength and consistency of associations across the contributing studies supported >90% likelihood for one of the novel loci to likely replicate in future studies (Table 3). Four lead variants were identified, linked to other 310 variants (Supplementary files 2 and 3). A gene-based association test revealed a significant association in *BAZ2B* and in previously known COVID-19 risk loci: *LZTFL1*, *XCR1*, *FYCO1*, *CCR9*, and *IFNAR2* (Supplementary file 4).

**Table 2.** Lead independent variants in the admixed AMR genome-wide association studies (GWAS) meta-analysis.

SNP rsID	chr:pos	EA	NEA	OR (95% CI)	p-Value	EAF cases	EAF controls	Nearest gene	Mamba PPR
*rs13003835*	2:159407982	T	C	1.20 (1.12–1.27)	3.66E-08	0.563	0.429	*BAZ2B*	0.30
*rs35731912*	3:45848457	T	C	1.65 (1.47–1.85)	6.30E-17	0.087	0.056	*LZTFL1*	0.95
*rs2477820*	6:41535254	A	T	0.84 (0.79–0.89)	1.89E-08	0.453	0.517	*FOXP4-AS1*	0.18
*rs77599934*	11:82906875	G	A	2.27 (1.7–3.04)	2.26E-08	0.016	0.011	*DDIAS*	0.95

EA: effect allele; NEA: noneffect allele; EAF: effect allele frequency in the SCOURGE study; PPR: posterior probability of replicability.

**Figure 2—figure supplement 1.**
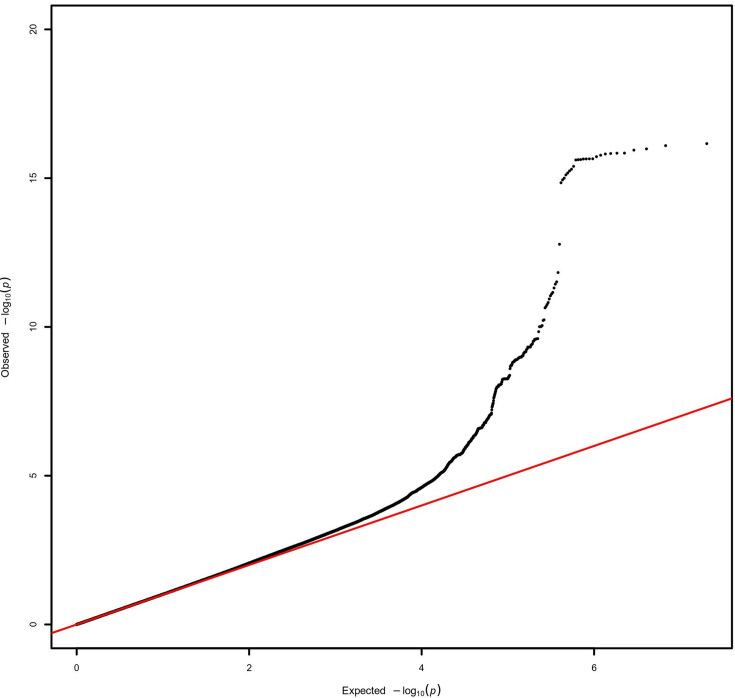
Quantile–quantile plot for the AMR genome-wide association studies (GWAS) meta-analysis. A lambda inflation factor of 1.015 was obtained.

**Table 3.** Novel variants in the SC-HGI_ALL_ and SC-HGI_3POP_ meta-analyses (with respect to HGIv7). Independent signals after LD clumping.

SNP rsID	chr:pos	EA	NEA	OR (95% CI)	p-Value	Nearest gene	Analysis
*rs76564172*	16:3892266	T	G	1.31 (1.19–1.44)	9.64E-09	*CREBBP*	SC-HGI_3POP_
*rs66833742*	19:4063488	T	C	0.94 (0.92–0.96)	1.89E-08	*ZBTB7A*	SC-HGI_3POP_
*rs66833742*	19:4063488	T	C	0.94 (0.92–0.96)	2.50E-08	*ZBTB7A*	SC-HGI_ALL_
*rs2876034*	20:6492834	A	T	0.95 (0.93–0.97)	2.83E-08	*CASC20*	SC-HGI_ALL_

EA: effect allele; NEA: non-effect allele.

**Figure 3.**
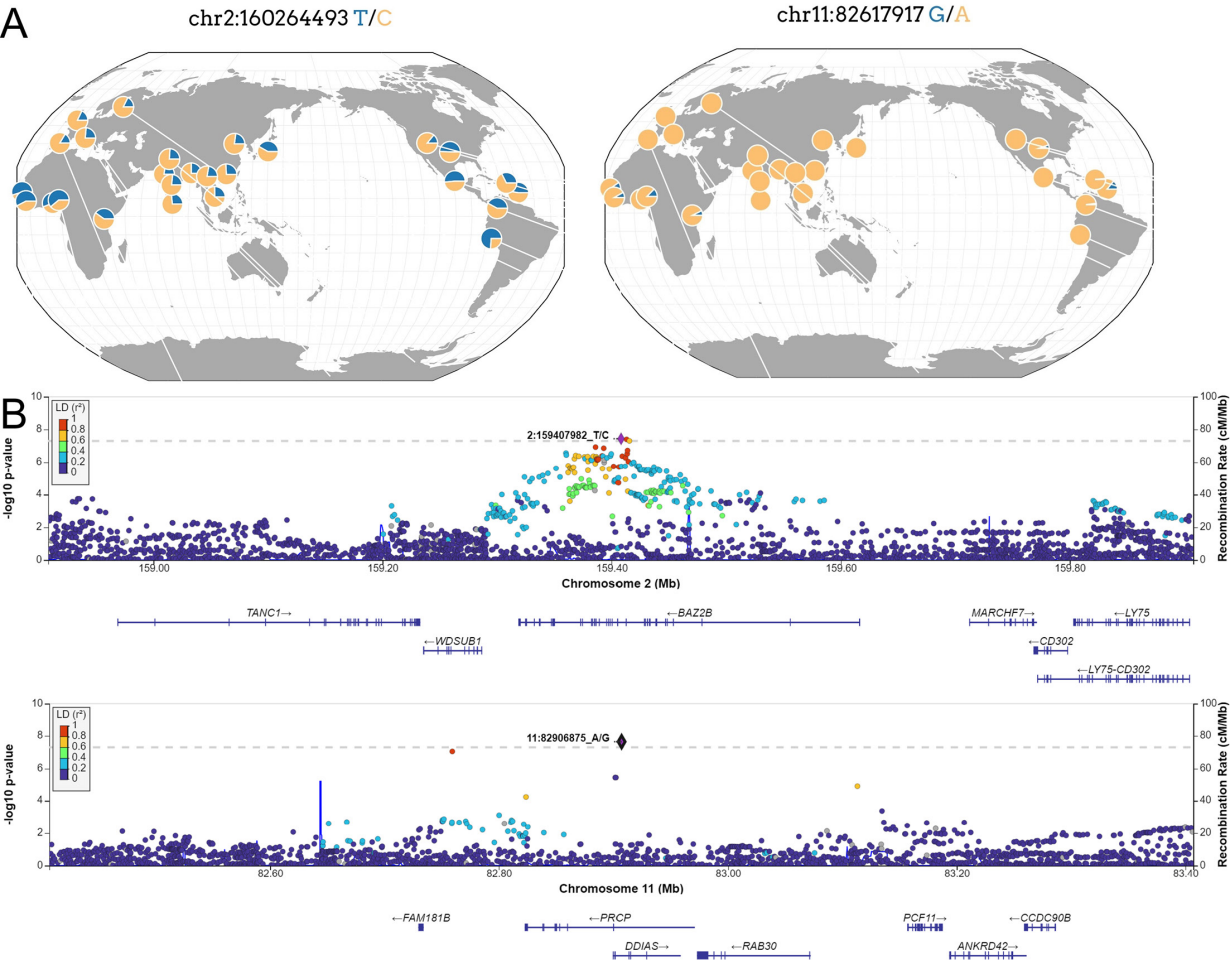
New loci associated with COVID-19 hospitalization in Admixed american populations. (**A**) Regional association plots for rs1003835 at chromosome 2 and rs77599934 at chromosome 11. (**B**) Allele frequency distribution across the 1000 Genomes Project populations for the lead variants rs1003835 and rs77599934. Retrieved from *The Geography of Genetic Variants Web* or GGV.

Located within the *BAZ2B* gene, the sentinel variant rs13003835 (Figure 3) is an intronic variant associated with an increased risk of COVID-19 hospitalization (odds ratio [OR]=1.20, 95% confidence interval [CI] = 1.12–1.27, p=3.66 × 10^–8^). This association was not previously reported in any GWAS of COVID-19 published to date. Interestingly, rs13003835 did not reach significance (p=0.972) in the COVID-19 HGI trans-ancestry meta-analysis including the five population groups (Kanai et al., 2023).

**Figure 3—figure supplement 1.**
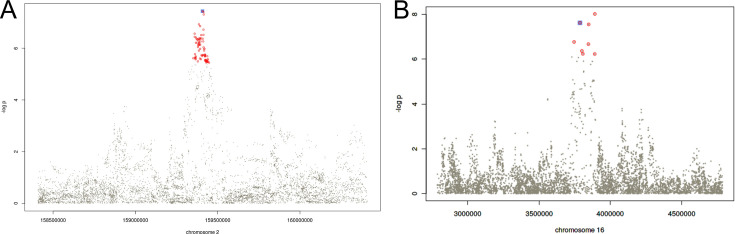
Regional association plots for the fine mapped loci in chromosomes 2 (**A**) and 16 (**B**). Colored in red, the variants allocated to the credible set at the 95% confidence according to the Bayesian fine mapping. In blue, the sentinel variant.

The other novel risk locus is led by the sentinel variant rs77599934 (Figure 3), a rare intronic variant located in chromosome 11 within *DDIAS* and associated with the risk of COVID-19 hospitalization (OR = 2.27, 95% CI = 1.70–3.04, p=2.26 × 10^–8^).

We also observed a suggestive association with rs2601183 in chromosome 15, which is located between *ZNF774* and *IQGAP1* (allele-G OR = 1.20, 95% CI = 1.12–1.29, p=6.11 × 10^–8^, see Supplementary file 2), which has not yet been reported in any other GWAS of COVID-19 to date.

The GWAS meta-analysis also pinpointed two significant variants at known loci, *LZTFL1* and *FOXP4*. The SNP rs35731912 was previously associated with COVID-19 severity in EUR populations (Degenhardt et al., 2022), and it was mapped to *LZTFL1*. While rs2477820 is a novel risk variant within the *FOXP4 gene*, it has a moderate LD (*r*^2^ = 0.295) with rs2496644, which has been linked to COVID-19 hospitalization (Kousathanas et al., 2022). This is consistent with the effects of LD in tag-SNPs when conducting GWAS in diverse populations.

None of the lead variants was associated with the comorbidities included in Table 1.

### Functional mapping of novel risk variants

Variants belonging to the lead loci were prioritized by positional and expression quantitative trait loci (eQTL) mapping with FUMA, resulting in 31 mapped genes (Supplementary file 5). Within the region surrounding the lead variant in chromosome 2, FUMA prioritized four genes in addition to *BAZ2B* (*PLA2R1*, *LY75*, *WDSUB1*, and *CD302*). rs13003835 (allele C) is an eQTL of *LY75* in the esophagus mucosa (NES = 0.27) and of *BAZ2B-AS* in whole blood (NES = 0.27), while rs2884110 (R^2^ = 0.85) is an eQTL of *LY75* in lung (NES = 0.22). As for the chromosome 11, rs77599934 (allele G) is in moderate-to-strong LD (r^2^ = 0.776) with rs60606421 (G deletion, allele -), which is an eQTL associated with a reduced expression of *DDIAS* in the lungs (NES = −0.49, allele -). The sentinel variant for the region in chromosome 16 is in perfect LD (*r*^2^ = 1) with rs601183, an eQTL of *ZNF774* in the lung.

#### Bayesian fine mapping

We performed different approaches to narrow down the prioritized loci to a set of most likely genes driving the associations. First, we computed credible sets at the 95% confidence level for causal variants and annotated them with VEP and V2G aggregate scoring. The 95% confidence credible set from the region of chromosome 2 around rs13003835 included 76 variants, which can be found in Supplementary file 6 and a regional plot is shown in Figure 3—figure supplement 1 (VEP and V2G annotations are included in Supplementary files 7 and 8). TheV2G score prioritized *BAZ2B* as the most likely gene driving the association. However, the approach was unable to converge allocating variants in a 95% confidence credible set for the region in chromosome 11.

#### Transcriptome-wide association study (TWAS)

Five novel genes, namely, *SLC25A37*, *SMARCC1*, *CAMP*, *TYW3*, and *S100A12* (Supplementary file 9), were found to be significantly associated in the cross-tissue TWAS. To our knowledge, these genes have not been reported previously in any COVID-19 TWAS or GWAS analyses published to date. In the single-tissue analyses, *ATP5O* and *CXCR6* were significantly associated in the lungs, *CCR9* was significantly associated in whole blood, and *IFNAR2* and *SLC25A37* were associated in lymphocytes.

Likewise, we carried out TWAS analyses using the models trained in the admixed populations. However, no significant gene pairs were detected in this case. The top 10 genes with the lowest p-values for each of the datasets (Puerto Ricans, Mexicans, African Americans, and pooled cohorts) are shown in Supplementary file 10. Although not significant, *KCNC3* was repeated in the four analyses, whereas *MAPKAPK3*, *NAPSA,* and *THAP5* were repeated in three out of four. Both *NAPSA* and *KCNC3* are located in the chromosome 19 and were reported in the latest HGI meta-analysis (Kanai et al., 2023).

**Figure 4.**
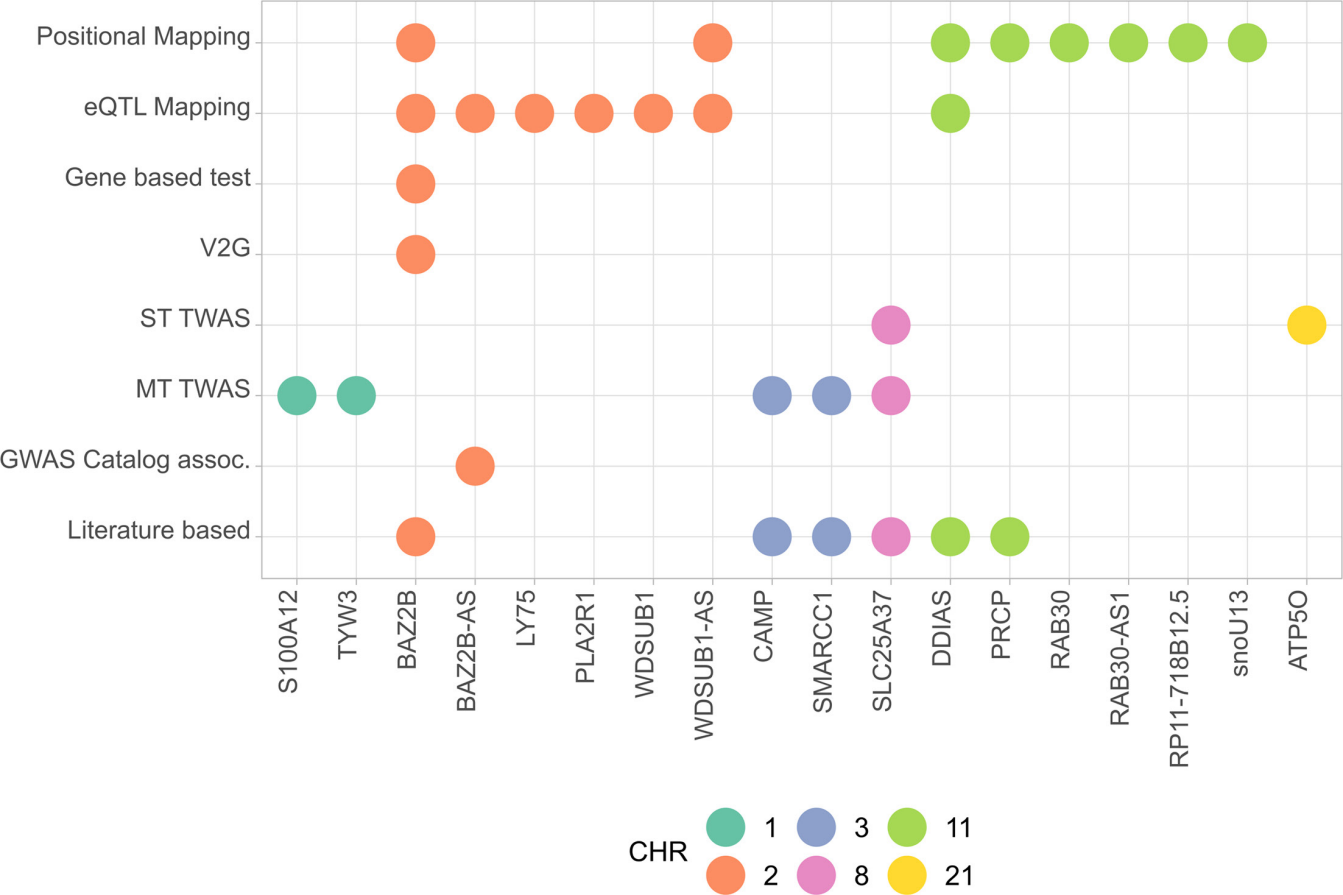
Summary of the results from gene prioritization strategies used for genetic associations in AMR populations. Genome-wide association studies (GWAS) catalog association for *BAZ2B-AS* was with FEV/FCV ratio. Literature-based evidence is further explored in ‘Discussion’.

All mapped genes from analyses conducted in AMR populations are shown in Figure 4, and associations for the two novel variants with expression are shown in Figure 4—figure supplement 1.

**Figure 4—figure supplement 1.**
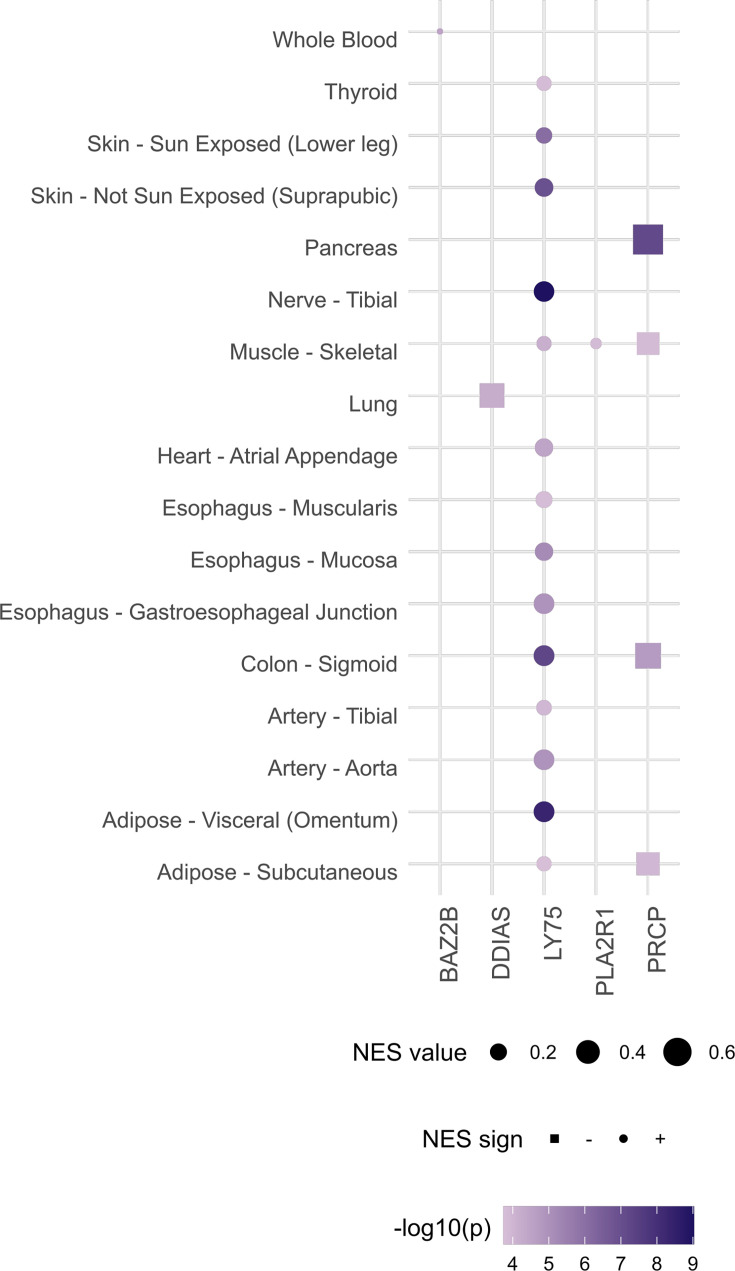
Gene‒tissue pairs for which either rs1003835 or rs60606421 are significant expression quantitative trait loci (eQTL) at false discovery rate (FDR) < 0.05 (data retrieved from https://gtexportal.org/home/snp/). rs1003835 (chromosome 2) maps to *BAZ2B*, *LY75*, and *PLA2R1* genes. As for the lead variant of chromosome 11, rs77599934, since it was not an eQTL, we used an LD proxy variant (rs60606421). *DDIAS* and *PRCP* genes map closely to this variant. NES and p-values correspond to the normalized effect size (and direction) of eQTL-gene associations and the p-value for the tissue, respectively.

### Genetic architecture of COVID-19 hospitalization in AMR populations

#### Allele frequencies of rs13003835 and rs77599934 across ancestries

Neither rs13003835 (*BAZ2B*) nor rs77599934 (*DDIAS*) were significantly associated in the COVID-19 HGI B2 cross-population or population-specific meta-analyses. Thus, we investigated their allele frequencies (AF) across populations and compared their effect sizes.

**Figure 5.**
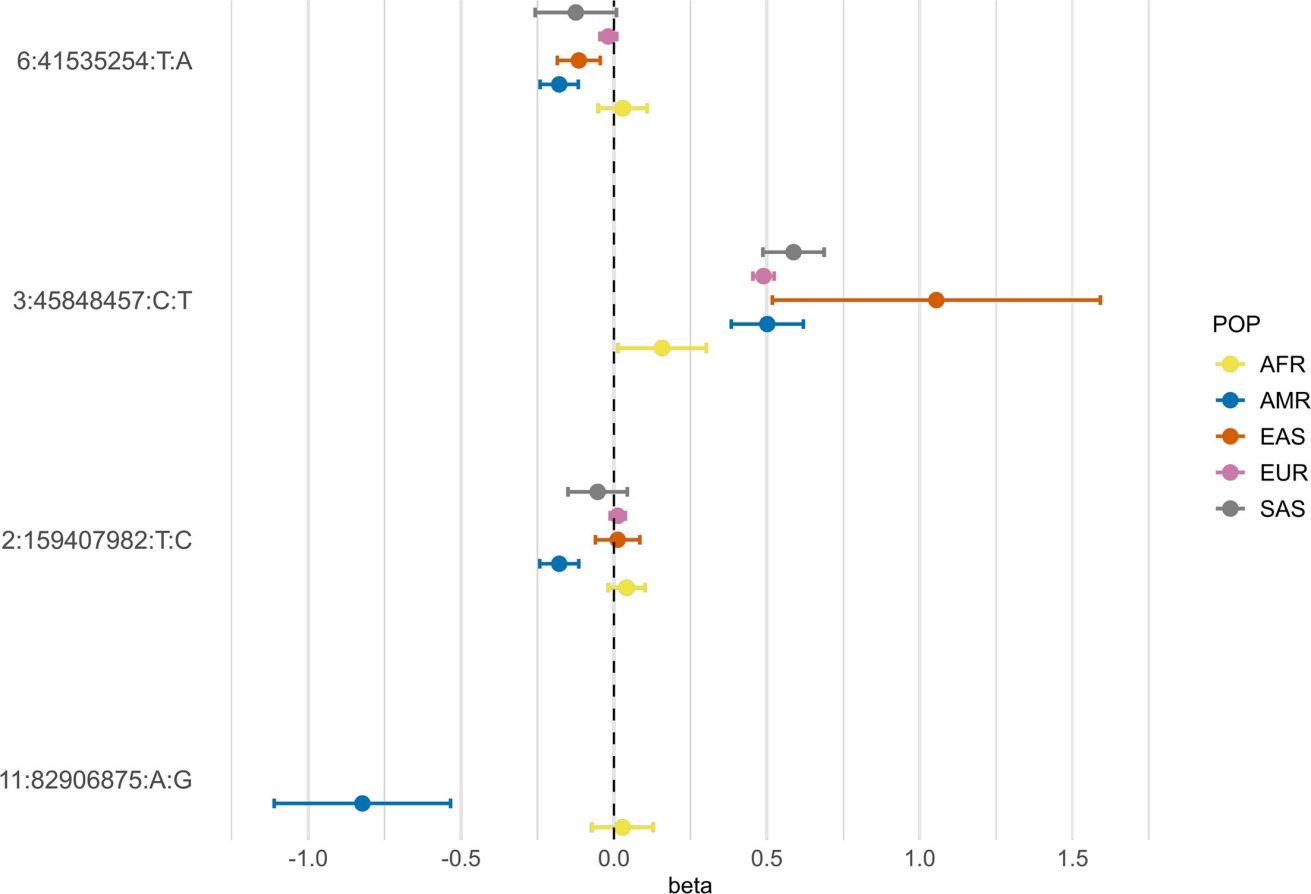
Forest plot showing effect sizes and the corresponding confidence intervals for the sentinel variants identified in the AMR meta-analysis across populations. All beta values with their corresponding CIs were retrieved from the B2 population-specific meta-analysis from the HGI v7 release, except for AMR, for which the beta value and IC from the HGI_AMR_-SCOURGE meta-analysis are represented.

According to gnomAD v3.1.2, the T allele at rs13003835 (*BAZ2B*) has an AF of 43% in admixed AMR groups, while AF is lower in the EUR populations (16%) and in the global sample (29%). Local ancestry inference (LAI) reported by gnomAD shows that within the Native American component, the risk allele T is the major allele, whereas it is the minor allele within the African and European LAI components. These large differences in AF might be the reason underlying the association found in AMR populations. However, when comparing effect sizes between populations, we found that they were in opposite directions between SAS-AMR and EUR-AFR-EAS and that there was large heterogeneity among them (Figure 5). We queried SNPs within 50 kb windows of the lead variant in each of the other populations that had p-value <0.01. The variant with the lowest p-value in the EUR population was rs559179177 (p=1.72 × 10^–4^), which is in perfect LD (r^2^ = 1) in the 1KGP EUR population with our sentinel variant (rs13003835), and in moderate LD r^2^ = 0.4 in AMR populations. Since this variant was absent from the AMR analysis, probably due to its low frequency, it could not be meta-analyzed. Power calculations revealed that the EUR analysis was underpowered for this variant to achieve genome-wide significance (77.6%, assuming an effect size of 0.46, EAF = 0.0027, and number of cases/controls as shown in the HGI website for B2-EUR). In the cross-population meta-analysis (B2-ALL), rs559179177 obtained a p-value of 5.9 × 10^–4^.

rs77599934 (*DDIAS*) had an AF of 1.1% for the G allele in the non-hospitalized controls (Table 2), in line with the recorded gnomAD AF of 1% in admixed AMR groups. This variant has the potential to be a population-specific variant, given the allele frequencies in other population groups, such as EUR (0% in Finnish, 0.025% in non-Finnish), EAS (0%) and SAS (0.042%), and its greater effect size over AFR populations (Figure 5). Examining the LAI, the G allele occurs at a 10.8% frequency in the African component, while it is almost absent in the Native American and European. Due to its low MAF, rs77599934 was not analyzed in the COVID-19 HGI B2 cross-population meta-analysis and was only present in the HGI B2 AFR population-specific meta-analysis, precluding the comparison (Figure 5). For this reason, we retrieved the variant with the lowest p-value within a 50 kb region around rs77599934 in the COVID-19 HGI cross-population analysis to investigate whether it was in moderate-to-strong LD with our sentinel variant. The variant with the smallest p-value was rs75684040 (OR = 1.07, 95% CI = 1.03–1.12, p=1.84 × 10^–3^). However, LD calculations using the 1KGP phase 3 dataset indicated that rs77599934 and rs75684040 were poorly correlated (r^2^ = 0.11). As for AFR populations, the variant with the lowest p-value was rs138860115 (p=8.3 × 10^–3^), but it was not correlated with the lead SNP of this locus.

#### Cross-population meta-analyses

We carried out two cross-ancestry inverse variance-weighted fixed-effects meta-analyses with the admixed AMR GWAS meta-analysis results to evaluate whether the discovered risk loci replicated when considering other population groups. In doing so, we also identified novel cross-population COVID-19 hospitalization risk loci.

First, we combined the SCOURGE Latin American GWAS results with the HGI B2 ALL analysis (Supplementary file 11). We refer to this analysis as the SC-HGI_ALL_ meta-analysis. Out of the 40 genome-wide significant loci associated with COVID-19 hospitalization in the last HGI release (Kanai et al., 2023), this study replicated 39, and the association was stronger than in the original study in 29 of those (Supplementary file 12). However, the variant rs13003835 located in *BAZ2B* did not replicate (OR = 1.00, 95% CI = 0.98–1.03, p=0.644).

In this cross-ancestry meta-analysis, we replicated two associations that were not found in HGIv7, albeit they were sentinel variants in the latest GenOMICC meta-analysis (Pairo-Castineira et al., 2023). We found an association at the *CASC20* locus led by the variant rs2876034 (OR = 0.95, 95% CI = 0.93–0.97, p=2.83 × 10^–8^). This variant is in strong LD with the sentinel variant of that study (rs2326788, r^2^ = 0.92), which was associated with critical COVID-19 (Pairo-Castineira et al., 2023). In addition, this meta-analysis identified the variant rs66833742 near *ZBTB7A* associated with COVID-19 hospitalization (OR = 0.94, 95% CI = 0.92–0.96, p=2.50 × 10^–8^). Notably, rs66833742 or its perfect proxy rs67602344 (r^2^ = 1) are also associated with upregulation of *ZBTB7A* in whole blood and in esophageal mucosa. This variant was previously associated with COVID-19 hospitalization (Pairo-Castineira et al., 2023).

In a second analysis, we also explored the associations across the defined admixed AMR, EUR, and AFR ancestral sources by combining through meta-analysis the SCOURGE Latin American GWAS results with the HGI studies in EUR, AFR, and admixed AMR and excluding those from EAS and SAS (Supplementary file 13). We refer to this as the SC-HGI_3POP_ meta-analysis. The association at rs13003835 (*BAZ2B*, OR = 1.01, 95% CI = 0.98–1.03, p=0.605) was not replicated, and rs77599934 near *DDIAS* could not be assessed, although the association at the *ZBTB7A* locus was confirmed (rs66833742, OR = 0.94, 95% CI = 0.92–0.96, p=1.89 × 10^–8^). The variant rs76564172 located near *CREBBP* also reached statistical significance (OR = 1.31, 95% CI = 1.25–1.38, p=9.64 × 10^–9^). The sentinel variant of the region linked to *CREBBP* (in the trans-ancestry meta-analysis) was also subjected to a Bayesian fine mapping (Supplementary file 6). Eight variants were included in the credible set for the region in chromosome 16 (meta-analysis SC-HGI_3POP_).

### Polygenic risk score models

Using the 49 variants associated with disease severity that are shared across populations according to the HGIv7, we constructed a PGS model to assess its generalizability in the admixed AMR (Supplementary file 14). First, we calculated the PGS for the SCOURGE Latin Americans and explored the association with COVID-19 hospitalization under a logistic regression model. The PGS model was associated with a 1.48-fold increase in COVID-19 hospitalization risk per every PGS standard deviation. It also contributed to explaining a slightly larger variance (∆R^2^ = 1.07%) than the baseline model.

Subsequently, we divided the individuals into PGS deciles and percentiles to assess their risk stratification. The median percentile among controls was 40, while in cases, it was 63. Those in the top PGS decile exhibited a 2.89-fold (95% CI = 2.37–3.54, p=1.29 × 10^–7^) greater risk compared to individuals in the deciles between 4 and 6 (corresponding to a score of the median distribution).

**Figure 6.**
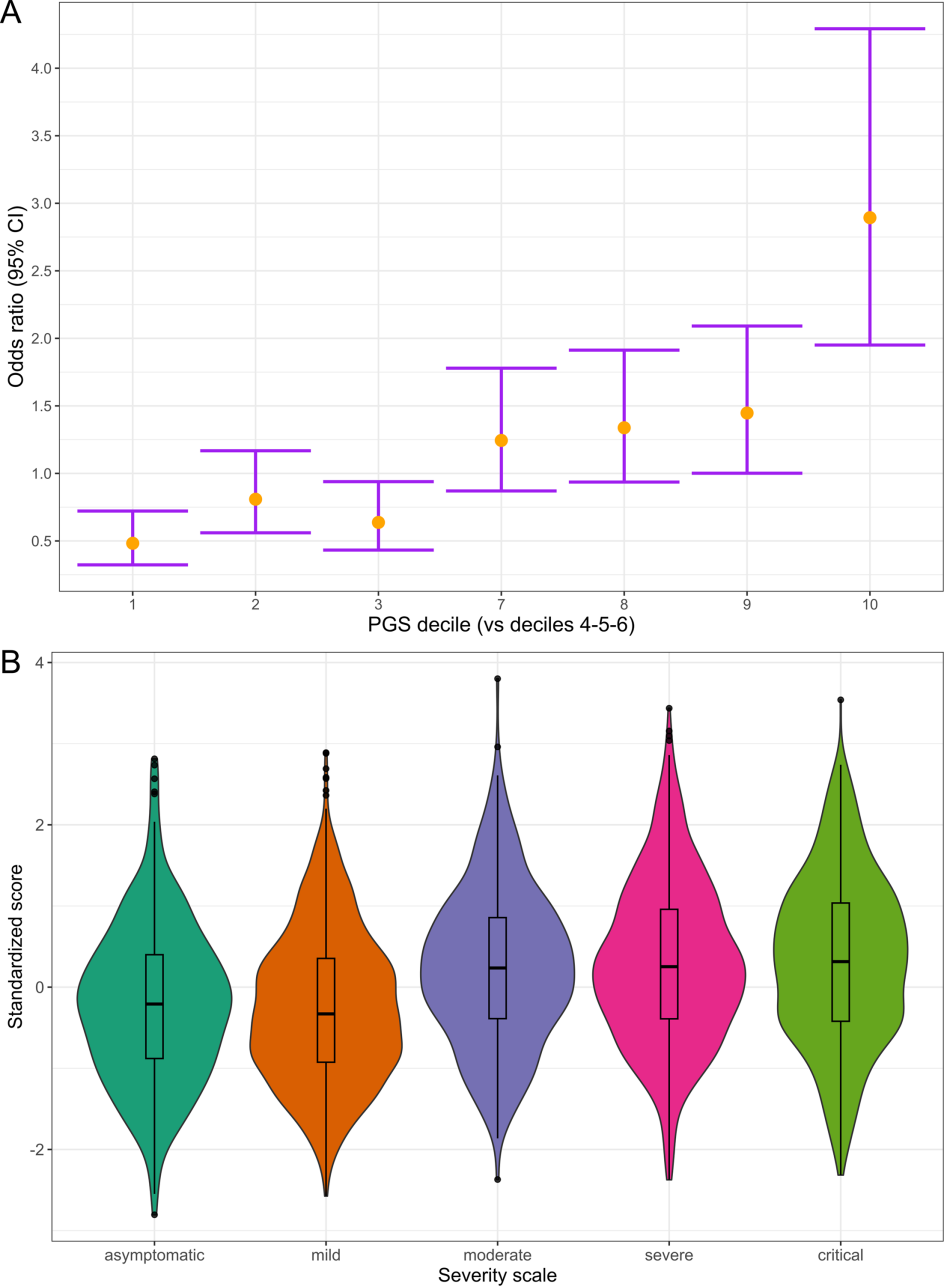
Polygenic risk distribution for COVID-19 hospitalization. (**A**) Polygenic risk stratified by polygenic risk score (PGS) deciles comparing each risk group against the lowest risk group (OR–95% CI). (**B**) Distribution of the PGS in each of the severity scale classes. 0, asymptomatic; 1, mild disease; 2, moderate disease; 3, severe disease; 4, critical disease.

We also examined the distribution of PGS across a five-level severity scale to further determine if there was any correspondence between clinical severity and genetic risk. Median PGS were lower in the asymptomatic and mild groups, whereas higher median scores were observed in the moderate, severe, and critical patients (Figure 6). We fitted a multinomial model using the asymptomatic class as a reference and calculated the OR for each category (Supplementary file 15), observing that the disease genetic risk was similar among asymptomatic, mild, and moderate patients. Given that the PGS was built with variants associated with critical disease and/or hospitalization and that the categories severe and critical correspond to hospitalized patients, these results underscore the ability of cross-ancestry PGS for risk stratification even in an admixed population.

## Discussion

We have conducted the largest GWAS meta-analysis of COVID-19 hospitalization in admixed AMR to date. While the genetic risk basis discovered for COVID-19 is largely shared among populations, trans-ancestry meta-analyses on this disease have primarily included EUR samples. This dominance of GWAS in Europeans and the subsequent bias in sample sizes can mask population-specific genetic risks (i.e., variants that are monomorphic in some populations) or be less powered to detect risk variants having higher allele frequencies in population groups other than Europeans. In this sense, after combining data from admixed AMR patients, we found two risk loci that were first discovered in a GWAS of Latin American populations. Interestingly, the sentinel variant rs77599934 in the *DDIAS* gene is a rare coding variant (~1% for allele G) with a large effect on COVID-19 hospitalization that is nearly monomorphic in most of the other populations. This has likely led to its exclusion from the cross-population meta-analyses conducted to date, remaining undetectable.

Fine mapping of the region harboring *DDIAS* did not reveal further information about which gene could be the more prone to be causal or about the functional consequences of the risk variant, but our sentinel variant was in strong LD with an eQTL that associated with reduced gene expression of *DDIAS* in the lung. *DDIAS,* known as damage-induced apoptosis suppressor gene, is itself a plausible candidate gene. It has been linked to DNA damage repair mechanisms: research has shown that depletion of *DDIAS* leads to an increase in ATM phosphorylation and the formation of p53-binding protein (53BP1) foci, a known biomarker of DNA double-strand breaks, suggesting a potential role in double-strand break repair (Brunette et al., 2019). Interestingly, a study found that infection by SARS-CoV-2 also triggered the phosphorylation of ATM kinase and inhibited repair mechanisms, causing the accumulation of DNA damage (Gioia et al., 2023). This gene has also been proposed as a potential biomarker for lung cancer after finding that it interacts with STAT3 in lung cancer cells, regulating IL-6 (Im et al., 2020; Im et al., 2023) and thus mediating inflammatory processes, while another study determined that its blockade inhibited lung cancer cell growth (Won et al., 2014). Another prioritized gene from this region was *PRCP*, an angiotensinase that shares substrate specificity with ACE2 receptor. It has been positively linked to hypertension and some studies have raised hypotheses on its role in COVID-19 progression, particularly in relation to the development of pro-thrombotic events (Angeli et al., 2023; Silva-Aguiar et al., 2020).

The risk region found in chromosome 2 harbors more than one gene. The lead variant rs13003835 is located within *BAZ2B*, and it increases the expression of the antisense *BAZ2B* gene in whole blood. *BAZ2B* encodes one of the regulatory subunits of the Imitation switch (ISWI) chromatin remodelers (Li et al., 2021) constituting the BRF-1/BRF-5 complexes with SMARCA1 and SMARCA5, respectively. Interestingly, it was discovered that *lnc-BAZ2B* promotes macrophage activation through regulation of *BAZ2B* expression. Its overexpression resulted in pulmonary inflammation and elevated levels of *MUC5AC* in mice with asthma (Xia et al., 2021). This variant was also an eQTL for *LY75* (encoding lymphocyte antigen 75) in the esophageal mucosa tissue. Lymphocyte antigen 75 is involved in immune processes through antigen presentation in dendritic cells and endocytosis (Mahnke et al., 2000) and has been associated with inflammatory diseases, representing a compelling candidate for the region. Increased expression of *LY75* has been detected within hours after infection by SARS-CoV-2 (Mitchell et al., 2013; Sims et al., 2013). It is worth noting that differences in AF for this variant suggest that analyses in AMR populations might be more powered to detect the association, supporting the necessity of population-specific studies.

A third novel risk region was observed on chromosome 15 between the *IQGAP1* and *ZNF774 genes*, although it did not reach genome-wide significance.

Secondary analyses revealed five TWAS-associated genes, some of which have already been linked to severe COVID-19. In a comprehensive multitissue gene expression profiling study (Gómez-Carballa et al., 2022), decreased expression of *CAMP* and *S100A8*/*S100A9* genes in patients with severe COVID-19 was observed, while another study detected the upregulation of *SCL25A37* among patients with severe COVID-19 (Policard et al., 2021). *SMARCC1* is a subunit of the SWI/SNF chromatin remodeling complex that has been identified as proviral for SARS-CoV-2 and other coronavirus strains through a genome-wide screen (Wei et al., 2021). This complex is crucial for *ACE2* expression and viral entry into the cell (Wei et al., 2023). However, it should be noted that using eQTL mostly from European populations such as those in GTEx could result in reduced power to detect associations.

To explore the genetic architecture of the trait among admixed AMR populations, we performed two cross-ancestry meta-analyses including the SCOURGE Latin American cohort GWAS findings. We found that the two novel risk variants were not associated with COVID-19 hospitalization outside the population-specific meta-analysis, highlighting the importance of complementing trans-ancestry meta-analyses with group-specific analyses. Notably, this analysis did not replicate the association at the *DSTYK* locus, which was associated with severe COVID-19 in Brazilian individuals with higher European admixture (Pereira et al., 2022). This lack of replication aligns with the initial hypothesis of that study suggesting that the risk haplotype was derived from European populations, as we reduced the weight of this ancestral contribution in our study by excluding those individuals.

Moreover, these cross-ancestry meta-analyses pointed to three loci that were not genome-wide significant in the HGIv7 ALL meta-analysis: a novel locus at *CREBBP* and two loci at *ZBTB7A* and *CASC20* that were reported in another meta-analysis. *CREBBP* and *ZBTB7A* achieved a stronger significance when considering only the EUR, AFR, and admixed AMR GIA groups. According to a recent study, elevated levels of the *ZBTB7A* gene promote a quasihomeostatic state between coronaviruses and host cells, preventing cell death by regulating oxidative stress pathways (Zhu et al., 2022). This gene is involved in several signaling pathways, such as B- and T-cell differentiation (Gupta et al., 2020). On a separate note, *CREBBP* encodes the CREB binding protein (CBP), which is involved in transcriptional activation and is known to positively regulate the type I interferon response through virus-induced phosphorylation of IRF-3 (Yoneyama et al., 1998). In addition, the CREBP/CBP interaction has been implicated in SARS-CoV-2 infection (Yang et al., 2023) via the cAMP/PKA pathway. In fact, cells with suppressed *CREBBP* gene expression exhibit reduced replication of the so-called Delta and Omicron SARS-CoV-2 variants (Yang et al., 2023).

We developed a cross-population PGS model, which effectively stratified individuals based on their genetic risk and demonstrated consistency with the clinical severity classification of the patients. Only a few polygenic scores were derived from COVID-19 GWAS data. Horowitz et al., 2022 developed a score using 6 and 12 associated variants (PGS ID: PGP000302) and reported an associated OR (top 10% vs rest) of 1.38 for risk of hospitalization in European populations, whereas the OR for Latin American populations was 1.56. Since their sample size and the number of variants included in the PGS were lower, direct comparisons are not straightforward. Nevertheless, our analysis provides the first results for a PRS applied to a relatively large AMR cohort, being of value for future analyses regarding PRS transferability.

This study is subject to limitations, mostly concerning sample recruitment and composition. The SCOURGE Latino American sample size is small, and the GWAS is likely underpowered. Another limitation is the difference in case‒control recruitment across sampling regions that, yet controlled for, may reduce the ability to observe significant associations driven by different compositions of the populations. In this sense, the identified risk loci might not replicate in a cohort lacking any of the parental population sources from the three-way admixture. Likewise, we could not explicitly control for socioenvironmental factors that could have affected COVID-19 spread and hospitalization rates, although genetic principal components are known to capture nongenetic factors. Finally, we must acknowledge the lack of a replication cohort. We used all the available GWAS data for COVID-19 hospitalization in admixed AMR in this meta-analysis due to the low number of studies conducted. Therefore, we had no studies to replicate or validate the results. These concerns may be addressed in the future by including more AMR GWAS in the meta-analysis, both by involving diverse populations in study designs and supporting research from countries in Latin America.

This study provides novel insights into the genetic basis of COVID-19 severity, emphasizing the importance of considering host genetic factors by using non-European populations, especially of admixed sources. Such complementary efforts can pin down new variants and increase our knowledge on the host genetic factors of severe COVID-19.

## Materials and methods

**Key resources table:** 

Reagent type (species) or resource	Designation	Source or reference	Identifiers	Additional information
Commercial assay or kit	Chemagic DNA Blood 100 kit	PerkinElmer Chemagen Technologies GmbH		
Software, algorithm	Axiom Analysis Suite	Thermo Fisher Scientific		Version 4.0.3.3
Software, algorithm	PLINK	Purcell et al., 2007; https://www.cog-genomics.org/plink/	RRID:SCR_001757	Version 1.9; v2
Software, algorithm	TOPMed Imputation Server	https://imputation.biodatacatalyst.nhlbi.nih.gov/		Version 2
Software, algorithm	ADMIXTURE	Alexander et al., 2009; https://dalexander.github.io/admixture/	RRID:SCR_001263	Version 1.3.0
Software, algorithm	SAIGEgds	Zheng and Davis, 2021; https://www.bioconductor.org/packages/release/bioc/html/SAIGEgds.html		Version 1.10.0
Software, algorithm	METAL	Willer et al., 2010; https://csg.sph.umich.edu/abecasis/metal/	RRID:SCR_002013	Version 2011-03-25
Software, algorithm	FUMA	Watanabe et al., 2017; https://fuma.ctglab.nl/	RRID:SCR_017521	Version 1.5.2
Software, algorithm	MAMBA	McGuire et al., 2021; https://github.com/dan11mcguire/mamba		Version 1
Software, algorithm	S-PrediXcan; S-MultiXcan	Barbeira et al., 2018; https://github.com/hakyimlab/MetaXcan	RRID:SCR_016739	Version 1
Software, algorithm	GTEx v8 mashr prediction models	https://predictdb.org/post/2021/07/21/gtex-v8-models-on-eqtl-and-sqtl/		
Other	GWAS Catalog	https://www.ebi.ac.uk/gwas/	RRID:SCR_012745	Section ‘Definition of the genetic risk loci and putative functional impact’

### GWAS in Latin Americans from SCOURGE

#### The SCOURGE Latin American cohort

A total of 3729 COVID-19-positive cases were recruited across five countries from Latin America (Mexico, Brazil, Colombia, Paraguay, and Ecuador) by 13 participating centers (Supplementary file 1) from March 2020 to July 2021. In addition, we included 1082 COVID-19-positive individuals recruited between March and December 2020 in Spain who either had evidence of origin from a Latin American country or showed inferred genetic admixture between AMR, EUR, and AFR (with <0.05% SAS/EAS). These individuals were excluded from a previous SCOURGE study that focused on participants with European genetic ancestries (Cruz et al., 2022). We used hospitalization as a proxy for disease severity and defined COVID-19-positive patients who underwent hospitalization as a consequence of the infection as cases and those who did not need hospitalization due to COVID-19 as controls.

Samples and data were collected with informed consent after the approval of the Ethics and Scientific Committees from the participating centers and by the Galician Ethics Committee Ref 2020/197. Recruitment of patients from IMSS (in Mexico City) was approved by the National Committee of Clinical Research from Instituto Mexicano del Seguro Social, Mexico (protocol R-2020-785-082).

Samples and data were processed following normalized procedures. The REDCap electronic data capture tool (Harris et al., 2009; Harris et al., 2019), hosted at Centro de Investigación Biomédica en Red (CIBER) from the Instituto de Salud Carlos III (ISCIII), was used to collect and manage demographic, epidemiological, and clinical variables. Subjects were diagnosed with COVID-19 based on quantitative PCR tests (79.3%) or according to clinical (2.2%) or laboratory procedures (antibody tests: 16.3%; other microbiological tests: 2.2%).

#### SNP array genotyping

Genomic DNA was obtained from peripheral blood and isolated using the Chemagic DNA Blood 100 kit (PerkinElmer Chemagen Technologies GmbH), following the manufacturer’s recommendations.

Samples were genotyped with the Axiom Spain Biobank Array (Thermo Fisher Scientific) following the manufacturer’s instructions in the Santiago de Compostela Node of the National Genotyping Center (CeGen-ISCIII; http://www.usc.es/cegen). This array contains probes for genotyping a total of 757,836 SNPs. Clustering and genotype calling were performed using Axiom Analysis Suite v4.0.3.3 software.

#### Quality control steps and variant imputation

A quality control (QC) procedure using PLINK 1.9 (Purcell et al., 2007) was applied to both samples and the genotyped SNPs. We excluded variants with a minor allele frequency (MAF) <1%, a call rate <98%, and markers strongly deviating from Hardy–Weinberg equilibrium expectations (p<1 × 10^–6^) with mid-p adjustment. We also explored the excess of heterozygosity to discard potential cross-sample contamination. Samples missing >2% of the variants were filtered out. Subsequently, we kept the autosomal SNPs, removed high-LD regions, and conducted LD pruning (windows of 1000 SNPs, with a step size of 80 and an r^2^ threshold of 0.1) to assess kinship and estimate the global ancestral proportions. Kinship was evaluated based on IBD values, removing one individual from each pair with PI_HAT > 0.25 that showed a Z0, Z1, and Z2 coherent pattern (according to the theoretical expected values for each relatedness level). Genetic principal components (PCs) were calculated with PLINK with the subset of LD pruned variants.

Genotypes were imputed with the TOPMed version r2 reference panel (GRCh38) using the TOPMed Imputation Server, and variants with Rsq < 0.3 or with MAF <1% were filtered out. A total of 4348 individuals and 10,671,028 genetic variants were included in the analyses.

#### Genetic admixture estimation

Global GIA, referred to the genetic similarity to the used reference individuals, was estimated with ADMIXTURE (Alexander et al., 2009) v1.3 software following a two-step procedure. First, we randomly sampled 79 European (EUR) and 79 African (AFR) samples from The 1000 Genomes Project (1KGP) (Auton et al., 2015) and merged them with the 79 Native American (AMR) samples from Mao et al., 2007 keeping the biallelic SNPs. LD-pruned variants were selected from this merge using the same parameters as in the QC. We then ran an unsupervised analysis with K = 3 to redefine and homogenize the clusters and to compose a refined reference for the analyses by applying a threshold of ≥95% of belonging to a particular cluster. As a result, 20 AFR, 18 EUR, and 38 AMR individuals were removed. The same LD-pruned variants data from the remaining individuals were merged with the SCOURGE Latin American cohort to perform supervised clustering and estimate admixture proportions. A total of 471 samples from the SCOURGE cohort with >80% estimated European GIA were removed to reduce the weight of the European ancestral component, leaving a total of 3512 admixed Latin American (AMR) subjects for downstream analyses.

#### Association analysis

The results for the SCOURGE Latin American GWAS were obtained by testing for COVID-19 hospitalization as a surrogate of severity. To accommodate the continuum of GIA in the cohort, we opted for a joint testing of all the individuals as a single study using a mixed regression model as this approach has demonstrated a greater power and sufficient control of population structure (Wojcik et al., 2019). The SCOURGE cohort consisted of 3512 COVID-19-positive patients: cases (n = 1625) were defined as hospitalized COVID-19 patients, and controls (n = 1887) were defined as non-hospitalized COVID-19-positive patients.

Logistic mixed regression models were fitted using the SAIGEgds (Zheng and Davis, 2021) package in R, which implements the two-step mixed SAIGE (Zhou et al., 2018) model methodology and the SPA test. Baseline covariables included sex, age (continuous), and the first 10 PCs. To account for potential heterogeneity in the recruitment and hospitalization criteria across the participating countries, we adjusted the models by groups of the recruitment areas classified into six categories: Brazil, Colombia, Ecuador, Mexico, Paraguay, and Spain. This dataset has not been used in any previously published GWAS of COVID-19.

### Meta-analysis of Latin American populations

The results of the SCOURGE Latin American cohort were meta-analyzed with the AMR HGI-B2 data, conforming our primary analysis. Summary results from the HGI freeze 7 B2 analysis corresponding to the admixed AMR population were obtained from the public repository (April 8, 2022: https://www.covid19hg.org/results/r7/), summing up 3077 cases and 66,686 controls from seven contributing studies. We selected the B2 phenotype definition because it offered more power, and the presence of population controls not ascertained for COVID-19 does not have a drastic impact on the association results.

The meta-analysis was performed using an inverse-variance weighting method in METAL (Willer et al., 2010). The average allele frequency was calculated, and variants with low imputation quality (Rsq < 0.3) were filtered out, leaving 10,121,172 variants for meta-analysis.

Heterogeneity between studies was evaluated with Cochran’s *Q*-test. The inflation of results was assessed based on a genomic control (lambda).

#### Replicability of associations

The model-based method MAMBA (McGuire et al., 2021) was used to calculate the posterior probabilities of replication for each of the lead variant (PPR; PP that an SNP has a non-zero replicable effect). We defined PPR <0.1 as a low posterior probability of replication, following the original paper, whereas those with a PPR >90% were considered consistent and likely to replicate in future studies. Variants with p<1 × 10^–05^ were clumped and combined with random pruned variants from the 1KGP AMR reference panel. Then, MAMBA was applied to the set of significant and non-significant variants.

Each of the lead variants was also tested for association with the main comorbidities in the SCOURGE cohort with logistic regression models (adjusted by the same base covariables as the GWAS).

### Definition of the genetic risk loci and putative functional impact

#### Definition of lead variant and novel loci

To define the lead variants in the loci that were genome-wide significant, LD-clumping was performed on the meta-analysis data using a threshold p-value<5 × 10^–8^, clump distance = 1500 kb, independence set at a threshold r^2^ = 0.1 and the SCOURGE cohort genotype data as the LD reference panel. Independent loci were deemed as a novel finding if they met the following criteria: (1) p-value<5 × 10^–8^ in the meta-analysis and p-value>5 × 10^–8^ in the HGI B2 ALL meta-analysis or in the HGI B2 AMR and AFR and EUR analyses when considered separately; (2) Cochran’s *Q*-test for heterogeneity of effects is <0.05/N_loci_, where N_loci_ is the number of independent variants with p<5 × 10^–8^; and (3) the nearest gene has not been previously described in the latest HGIv7 update.

#### Annotation and initial mapping

Functional annotation was performed with FUMA (Watanabe et al., 2017) for those variants with a p-value<5 × 10^–8^ or in moderate-to-strong LD (r^2^ > 0.6) with the lead variants, where the LD was calculated from the 1KGP AMR panel. Genetic risk loci were defined by collapsing LD blocks within 250 kb. Then, genes, scaled CADD v1.4 scores, and RegulomeDB v1.1 scores were annotated for the resulting variants with ANNOVAR in FUMA (Watanabe et al., 2017). Gene-based analysis was also performed using MAGMA (de Leeuw et al., 2015) as implemented in FUMA under the SNP-wide mean model using the 1KGP AMR reference panel. Significance was set at a threshold p<2.66 × 10^–6^ (which assumes that variants can be mapped to a total of 18,817 genes).

FUMA allowed us to perform initial gene mapping by two approaches: (1) positional mapping, which assigns variants to genes by physical distance using 10 kb windows; and (2) eQTL mapping based on GTEx v.8 data from whole blood, lungs, lymphocytes, and esophageal mucosa tissues, establishing a false discovery rate (FDR) of 0.05 to declare significance for variant–gene pairs.

Subsequently, to assign the variants to the most likely gene driving the association, we refined the candidate genes by fine mapping the discovered regions.

#### Bayesian fine-mapping

To conduct a Bayesian fine mapping, credible sets for the genetic loci considered novel findings were calculated on the results from each of the three meta-analyses to identify a subset of variants most likely containing the causal variant at the 95% confidence level, assuming that there is a single causal variant and that it has been tested. We used *corrcoverage* (https://cran.rstudio.com/web/packages/corrcoverage/index.html) for R to calculate the posterior probabilities of the variant being causal for all variants with an r^2^ > 0.1 with the leading SNP and within 1 Mb except for the novel variant in chromosome 19, for which we used a window of 0.5 Mb. Variants were added to the credible set until the sum of the posterior probabilities was ≥0.95.

#### VEP and V2G annotation

We used the Variant-to-Gene (V2G) score to prioritize the genes that were most likely affected by the functional evidence based on eQTL, chromatin interactions, in silico functional predictions, and distance between the prioritized variants and transcription start site (TSS), based on data from the Open Targets Genetics portal (Ghoussaini et al., 2021). Details of the data integration and the weighting of each of the datasets are described in detail at https://genetics-docs.opentargets.org/our-approach/data-pipeline. V2G is a score for ranking the functional genomics evidence that supports the connection between variants and genes (the higher the score the more likely the variant to be functionally implicated on the assigned gene). We used VEP release 111 (https://www.ensembl.org/info/docs/tools/vep/index.html; accessed April 10, 2024; McLaren et al., 2016) to annotate the following: gene symbol, function (exonic, intronic, intergenic, non-coding RNA, etc.), impact, feature type, feature, and biotype.

We queried the GWAS catalog (date of accession: 1/07/2024) for evidence of association of each of the prioritized genes with traits related to lung diseases or phenotypes. Lastly, those which were linked to COVID-19, infection, or lung diseases in the revised literature were classified as ‘literature evidence’.

### Transcription-wide association studies

TWAS were conducted using the pretrained prediction models with MASHR-computed effect sizes on GTEx v8 datasets (Barbeira et al., 2019a; Barbeira et al., 2021). The results from the Latin American meta-analysis were harmonized and integrated with the prediction models through S-PrediXcan (Barbeira et al., 2018) for lung, whole blood, lymphocyte, and esophageal mucosal tissues. Statistical significance was set at p-value<0.05 divided by the number of genes that were tested for each tissue. Subsequently, we leveraged results for all 49 tissues and ran a multitissue TWAS (S-MultiXcan) to improve the power for association, as demonstrated recently (Barbeira et al., 2019b). TWAS was also performed using recently published gene expression datasets derived from a cohort of African Americans, Puerto Ricans, and Mexican Americans (GALA II-SAGE) (Kachuri et al., 2023).

### Cross-population meta-analyses

We conducted two additional meta-analyses to investigate the ability of combining populations to replicate our discovered risk loci. This methodology enabled the comparison of effects and the significance of associations in the novel risk loci between the results from analyses that included or excluded other population groups.

The first meta-analysis comprised the five populations analyzed within HGI (B2-ALL). Additionally, to evaluate the three GIA components within the SCOURGE Latin American cohort (Bryc et al., 2010), we conducted a meta-analysis of the admixed AMR, EUR, and AFR cohorts (B2). All summary statistics were retrieved from the HGI repository. We applied the same meta-analysis methodology and filters as in the admixed AMR meta-analysis.

### Cross-population polygenic risk score

A PGS for critical COVID-19 was derived by combining the variants associated with hospitalization or disease severity that have been discovered to date. We curated a list of lead variants that were (1) associated with either severe disease or hospitalization in the latest HGIv7 release (Kanai et al., 2023) (using the hospitalization weights) or (2) associated with severe disease in the latest GenOMICC meta-analysis (Pairo-Castineira et al., 2023) that were not reported in the latest HGI release. A total of 48 markers were used in the PGS model (see Supplementary file 13) since two variants were absent from our study.

Scores were calculated and normalized for the SCOURGE Latin American cohort with PLINK 1.9. This cross-ancestry PGS was used as a predictor for hospitalization (COVID-19-positive patients who were hospitalized vs COVID-19-positive patients who did not necessitate hospital admission) by fitting a logistic regression model. Prediction accuracy for the PGS was assessed by performing 500 bootstrap resamples of the increase in the pseudo-R^2^. We also divided the sample into deciles and percentiles to assess risk stratification. The models were fit for the dependent variable adjusting for sex, age, the first 10 PCs, and the sampling region (in the admixed AMR cohort) with and without the PGS, and the partial pseudo-R^2^ was computed and averaged among the resamples.

A clinical severity scale was used in a multinomial regression model to further evaluate the power of this cross-ancestry PGS for risk stratification. These severity strata were defined as follows: (0) asymptomatic; (1) mild, that is, with symptoms, but without pulmonary infiltrates or need of oxygen therapy; (2) moderate, that is, with pulmonary infiltrates affecting <50% of the lungs or need of supplemental oxygen therapy; (3) severe disease, that is, with hospital admission and PaO_2_ <65 mmHg or SaO_2_ <90%, PaO_2_/FiO_2_<300, SaO_2_/FiO_2_<440, dyspnea, respiratory frequency ≥22 bpm, and infiltrates affecting >50% of the lungs; and (4) critical disease, that is, with an admission to the ICU or need of mechanical ventilation (invasive or noninvasive).

## Data Availability

Summary statistics from the SCOURGE Latin American GWAS and the analysis scripts are available from the public repository https://github.com/CIBERER/Scourge-COVID19 (copy archived at CIBERER, 2024). The following previously published datasets were used: ConsortiumGTEx 2020GTEx V8GTEx PortalSingle-Tissue cis-QTL Consortium Genomes Project 20161000 Genomes Phase 3PLINK 2.0 Resources1000 Genomes phase 3 COVID-19 Host Genetics Initiative 2022COVID19-hg GWAS meta-analyses round 7COVID-19 hg repositoryr7

## Additional files

Supplementary file 1. Participating centers.

Supplementary file 2. Independent variants with p-value<1 × 10^–05^ in the SC-HGI_AMR GWAS meta-analysis (hg38). EA: effect allele; NEA: non-effect allele; EAF: effect allele frequency; EAF_avg: averaged effect allele frequency; FreqSE: standard error of averaged effect allele frequency; SCOURGE_AMR: SCOURGE Latin-America; HGIB2_AMR: HGI meta-analysis of AMR studies.

Supplementary file 3. Annotated SNPs in moderate-to-strong LD with lead SNPs of the genome-wide significant loci in the SC-HGI_AMR GWAS meta-analysis, with ANNOVAR. NEA: non-effect allele; EA: effect allele; r^2^: maximum r^2^ of the SNP with one of the independent SNPs; IndSigSNP: the independent SNP which has the maximum r^2^ value with the SNP; dist: distance to the nearest gene; func: functional consequence of the SNP on the gene; CADD: CADD score; RDB: RegulomeDB score; minChrState: the minimum 15-core chromatin state across 127 tissues/cell types; commonChrState: the most common 15-core chromatin state across 127 tissues/cell types; posMapFilt: 1 if the SNP was used for positional mapping, 0 otherwise; eqtlMapFilt: 1 if the SNP was used for eQTL mapping, 0 otherwise.

Supplementary file 4. Results from the MAGMA gene-based analysis in the SC-HGI_AMR GWAS meta-analysis (hg37). NSNPS: number of SNPs in the gene; NPARAM: the number of relevant parameters used in the model; ZSTAT: z statistics.

Supplementary file 5. Prioritized genes by eQTL and positional mapping by FUMA in the SC-HGI_AMR GWAS meta-analysis results (hg37). HUGO: HGNC gene symbol; pLI: pLI score from ExAC database, probability of being intolerant to loss of function (higher the score, higher the intolerance); ncRVIS: non-coding residual variation intolerance score (higher the score, higher intolerance to non-coding variation); posMapSNPs: number of SNPs mapped by positional mapping; posMapMaxCADD: the maximum CADD score of mapped SNPs by positional mapping; eqtlMapSNPS: the number of SNPs mapped to the genes based on eQTL mapping; eqtlMapminP: the minimum eQTL p-value of mapped SNPs; eqtlMapminQ: the minimum eQTL FDR of mapped SNPs; eqtlMapts: tissue of mapped eQTLs; eqtlDirection: consequential direction of mapped eQTL SNPs after aligning the risk alleles; minGwasP: minimum GWAS p-value of mapped eQTLs; IndSigSNPs: independent SNPs that are in LD with the mapped SNPs.

Supplementary file 6. Fine-mapped credible set derived with corrcoverage (95%) for the associated region in chromosome 2 (BAZ2B).

Supplementary file 7. VEP annotations for the variants included in the fine-mapped credible sets for the novel associated loci in chromosome 2 (hg38).

Supplementary file 8. V2G scores for the variants included in the fine-mapped credible sets in the novel risk loci from chromosomes 2 and 16 (hg38). Shaded in green, the prioritized gene by the V2G score.

Supplementary file 9. MultiXcan results for the SC-HGI_AMR GWAS meta-analysis. N: number of tissues available for the gene; n_indep: number of independent components of variation kept among the tissues' predictions; p_i_best: best p-value of single tissue S-prediXcan association; t_i_best: name of best single tissue S-prediXcan association; p_i_worst: worst p-value of single tissue S-prediXcan association; t_i_worst: name of worst single tissue S-prediXcan association; eigen_max: eigenvalue of the top independent component in the SVD decomposition of predicted expression correlation; eigen_min: eigenvalue of the last independent component in the SVD decomposition of predicted expression correlation; eigen_min_kept: eigenvalue of the smallest independent component that was kept in the SVD decomposition of predicted expression correlation; z_min: minimum z-score among single-tissue S-prediXcan associations; z_max: maximum z-score among single-tissue S-prediXcan associations; z_mean: mean z-score among single tissue S-prediXcan associations; z_sd: standard deviation of the mean z-score among single-tissue S-prediXcan associations; tmi: trace of T*T', where T is the correlation of predicted expression levels for different tissues multiplied by its SVD pseudo-inverse and is an estimate for the number of independent components of variation in predicted expression across tissues.

Supplementary file 10. Top 10 genes for the TWAS trained with the GALA II-SAGE models in admixed Americans. Bonferroni correction thresholds: Pooled p<4.19E-06; PR p<4.99E-06; MX p<5.19E-06; AA p<4.67E-06. Var_g: variance of the gene expression; pred_perf_r2: cross-validated R^2^ of tissue model’s correlation to gene’s measured transcriptome; pref_perf_qval: qval of tissue model’s correlation to gene’s measured transcriptome; n_snps_used: number of snps from GWAS used in S-prediXcan analysis; n_snp_in_cov: number of snps in the covariance matrix; n_snps_in_model: number of snps in the model; best_gwas_p: the highest p-value from GWAS snps used in this model; largest_weight: the largest weight in this model.

Supplementary file 11. Independent variants with p-value<1e-05 in the SC-HGI_ALL GWAS meta-analysis (hg38). EA: effect allele; NEA: non-effect allele; EAF_avg: averaged effect allele frequency; FreqSE: standard error of averaged effect allele frequency.

Supplementary file 12. Results of the 40 lead variants associated with COVID-19 hospitalization in the HGIv7 (hg38). SC-HGI_ALL: meta-analysis SCOURGE-HGI_ALL; SC-HGI_AMR: meta-analysis SCOURGE-HGI_AMR; SC-HGI_3POP: meta-analysis SCOURGE-HGI_3POP.

Supplementary file 13. Independent variants with p-value<1e-05 in the SC-HGI_3POP GWAS meta-analysis (hg38). EA: effect allele; NEA: non-effect allele; EAF_avg: average effect allele frequency; FreqSE: standard error of averaged effect allele frequency.

Supplementary file 14. Instruments used in the polygenic risk score model (hg38).

Supplementary file 15. Multinomial regression results. Reference class for the multinomial regression is ‘asymptomatic’.

MDAR checklist
